# Insights into the Natural and Treatment Courses of Hepatitis B in Children: A Retrospective Study

**DOI:** 10.3390/biomedicines12071585

**Published:** 2024-07-17

**Authors:** Lorenza Forna, Laura Bozomitu, Ancuta Lupu, Vasile Valeriu Lupu, Camelia Cojocariu, Carmen Anton, Irina Girleanu, Ana Maria Singeap, Cristina Maria Muzica, Anca Trifan

**Affiliations:** 1 Pediatrics–“Sf. Maria” Clinical Emergency Children’s Hospital, 700309 Iași, Romania; lorenza.donea@yahoo.ro (L.F.); laura.bozomitu@gmail.com (L.B.); anca_ign@yahoo.com (A.L.); valeriulupu@yahoo.com (V.V.L.); 2 Faculty of General Medicine, University of Medicine and Pharmacy “Gr. T. Popa”, 700115 Iași, Romania; cojocariu.salloum@umfiasi.ro (C.C.); carmen.anton@umfiasi.ro (C.A.); irina.girleanu@umfiasi.ro (I.G.); ana.singeap@umfiasi.ro (A.M.S.); anca.trifan@umfiasi.ro (A.T.); 3 Department of Clinical Gastroenterology, “Sf. Spiridon” Clinical Emergency Hospital, 700111 Iași, Romania

**Keywords:** children, HBV infection, spontaneous seroconversion, natural history

## Abstract

Chronic Hepatitis B virus (HBV) infection in children remains a significant public health challenge. The natural history and treatment outcomes of HBV can vary widely, influencing management strategies. This retrospective study was conducted in Northeast Romania and involved a cohort of 148 pediatric patients diagnosed with chronic viral Hepatitis B. Of these, 59 children underwent antiviral treatment while 89 were not treated. One of the main objectives was the rate of HBeAg (Hepatitis B-e antigen) seroconversion, a marker of disease progression and response to therapy. Among the treated group, 26 children (44%) achieved HBeAg seroconversion following therapy. In contrast, 44 of the untreated children (49%) experienced spontaneous HBeAg seroconversion, indicating a substantial rate of natural resolution within this population subset. The findings highlight a significant proportion of spontaneous seroconversion in untreated pediatric patients, suggesting a potential re-evaluation of treatment criteria and timing for children with chronic HBV infection. The comparable rates of seroconversion between treated and untreated cohorts underscore the need for individualized treatment approaches based on a combination of virological, biochemical, and clinical parameters. Further studies are required to refine management strategies to optimize long-term outcomes in pediatric HBV infections.

## 1. Introduction

Chronic Hepatitis B is the precursor stage to cirrhosis and the 10th leading cause of death globally. In 2022, the World Health Organization estimated that approximately 254 million people were chronically infected with the hepatitis B virus (HBV), resulting in over 800,000 deaths [1]. In 2016, the World Health Organization adopted a resolution to eliminate viral hepatitis as a public health threat by 2030 [2].

Regarding the pediatric population under the age of 5, it was estimated that the prevalence has decreased to 1.3% (2017) from 4.7% during the period spanning 1980–2000s [3]. However, Africa remains the main region with a prevalence of 3% [4]. It is also known that approximately 3–5% of children with chronic HBV infection develop cirrhosis, and 0.01–0.03% develop hepatocellular carcinoma [4]. HBV can be transmitted vertically, perinatally, or horizontally through sexual contact, contaminated blood, transplants, tissues, biological products, dental procedures, or other interventions with improperly sterilized instruments [5]. In the pediatric population, transmission is predominantly vertical and intrafamilial. In 2015, the global vaccination rate at birth for the Hepatitis B vaccine was 39%, with the highest coverage in the Western Pacific and Americas regions [6]. This remains a concern and underscores the notion that immunization at birth should be the first step in the prophylaxis of Hepatitis B virus infection [2,6].

In the general population, approximately 70% of HBV infection cases are asymptomatic, whereas 30% associate jaundice as the main clinical presentation [7]. Fulminant liver failure occurs in 0.1–0.5% of patients [4,7]. The incubation period varies between 1 and 4 months. During the prodromal period, nonspecific symptoms such as nausea, decreased appetite, fatigue, pain in the right hypochondrium, or jaundice may be present.

Chronic infection with HBV is defined by the presence of HBsAg (Hepatitis B surface antigen) in two successive tests over a period of at least 6 months. Although the rate of chronicity in newborns is 95%, this disease often presents mildly in childhood, with many children being asymptomatic. The characteristics of each phase are reported in Table 1. 

The need for treatment is unquestionable, with its purpose being to suppress viral replication and prevent the disease’s progression to cirrhosis or hepatocellular carcinoma. For establishing disease management, ALT (Alanine Aminotransferase) levels must be evaluated for a minimum of 6 months if they remain elevated (1.5 times the normal value) or 12 months for those with negative HBe antigen before considering antiviral therapy to avoid overlapping with the moment of seroconversion in the HBe system [8]. The importance of follow-up is undeniable, both for inactive patients who are at risk of disease progression to cirrhosis (7.8% over 25 years), hepatocellular carcinoma (2.2%), or chronic hepatitis with negative HBe antigen (15–24%), as well as reactivation of HBe antigen (1–4%), and also for patients who have developed anti-HBs antibodies, given that reactivation of the disease can occur in certain cases, sometimes with fulminant progression [8].

The main objectives of this study were the evaluation of the natural history of chronic HBV infection and HBeAg rate seroconversion, a marker of disease progression and response to therapy.

## 2. Materials and Method

In this retrospective study, we included data from a tertiary center in north-eastern Romania, encompassing a total of 654 children tested for HBsAg, a total of 148 pediatric patients diagnosed with chronic HBV infection. 

To track the progression and predict outcomes, a longitudinal discriminant analysis algorithm was employed, including HBeAg seroconversion rates. This approach facilitated a detailed evaluation of disease progression and response to treatment, shedding light on the natural history of chronic Hepatitis B in children and emphasizing the critical role of HBeAg seroconversion in predicting long-term health outcomes. This study includes interim results from PhD research investigating the natural history and post-treatment evolution of HBV among children admitted to the ‘Sf. Maria’ Hospital in Iasi from 1 January 2011 to 31 December 2019. Chronic HBV infection was identified by the persistence of hepatitis B surface antigen (HBsAg) for at least 6 months.

The criteria for inclusion were as follows:

- Individuals aged between 0 and 18 years at the time of their first pediatric consultation for chronic HBV infection.
- Detection of hepatitis B surface antigen (HBsAg) persisting for a minimum of six months.

Concomitant infections with human immunodeficiency virus (HIV), hepatitis C virus (HCV), or hepatitis D virus (HDV) and lack of initial data were considered criteria for exclusion.

Data collected from patient records included demographic information, reasons for hospitalization, clinical and paraclinical data necessary for staging the disease, antiviral treatment (if initiated), and the patient’s progress under treatment, along with HBe seroconversion (spontaneous/post-treatment).

Statistical Analysis: The statistical evaluations were conducted using SPSS version 29.0. Qualitative data were analyzed using frequency distributions, while quantitative data were assessed with descriptive statistics, including means and standard deviations. A significance level was set at *p* < 0.05.

## 3. Results

**Figure 1:**
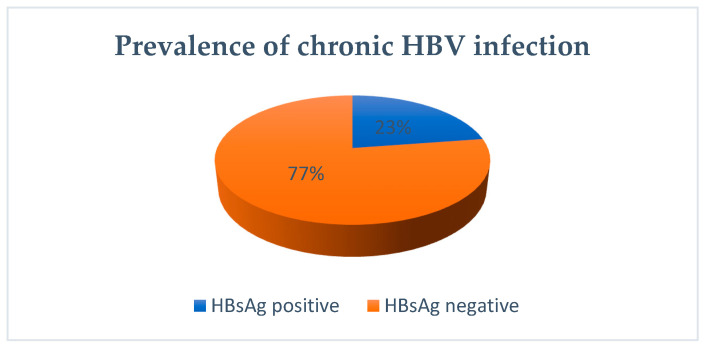
Prevalence of chronic HBV infection in study tested group.

The prevalence in the study cohort (23%) (Figure 1) is undoubtedly higher than in the general population, clearly due to the patient referrals in a hospital setting, specifically within the specialty of Pediatric Gastroenterology. Globally, for children under 5 years old, the prevalence has decreased to 1.3% compared to 4% in the years spanning 1980–2000. Current data do not provide information on the prevalence of chronic viral hepatitis B infection in children at the national or regional level. This study is the first of substantial significance both temporally (from 2011 to 2019) and in terms of its affiliation with a regional university center, Saint Mary’s Hospital being the first major pediatric hospital in Romania, with referrals from all the counties of Romanian N-E region and beyond.

### 3.1. Natural Evolution

All stages of HBV infection were detected among the patients. Most patients (49.3%) were diagnosed at the stage of chronic viral hepatitis B HBeAg positive, followed by HBV infection HBeAg positive (27.7%). Most patients with HBV hepatitis HBeAg- were from age groups 6–10 years and 15–18 years (54.6%). Most patients with HBV hepatitis HBeAg+ were from the age group 0–1 years (26%) followed by 2–5 years (23.3%). Most patients with HBV infection HBeAg- (34.8%) as well as HBeAg+ (29.3%) were from the age group 2–5 years (Table 2).

Of the patients studied, 56 (37.83%) were asymptomatic, and 92 (62.16%) were symptomatic (Table 3). Moreover, in our study, among those with HBeAg+ infection, 56.1% exhibited no specific signs or symptoms, with similar proportions found among those with HBeAg- infection. However, 75.3% of those with chronic HBeAg+ hepatitis displayed clinical symptoms and signs such as diffuse abdominal pain in the right hypochondrium or epigastrium, nausea, vomiting, loss of appetite, and physical asthenia. Additionally, 18 patients presented with hepatomegaly at onset, 6 were admitted with epistaxis, and 28 were hospitalized during a respiratory tract infection episode, experiencing symptoms such as cough, rhinorrhea, or odynophagia. It should be noted that among those in the HBeAg+ hepatitis stage, the majority experienced loss of appetite, followed by hepatomegaly (20.5%) and abdominal pain (15.1%). Children with HBeAg- or HBeAg+ infection predominantly complained of loss of appetite, while abdominal pain and hepatomegaly were common in HBeAg- hepatitis (Table 4).

**Figure 2:**
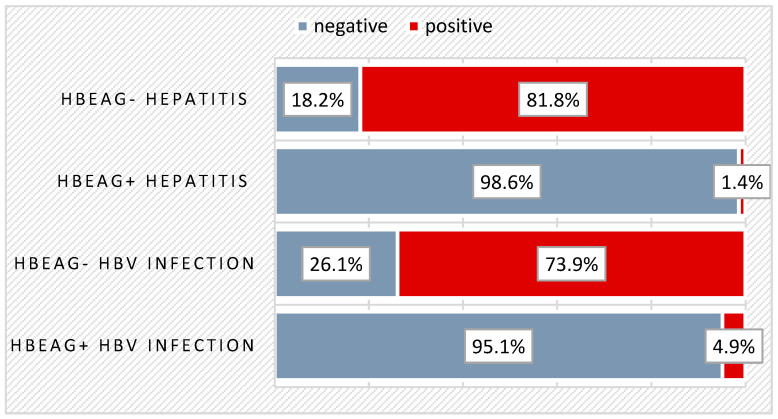
Distribution of patients based on the presence of anti-HBe antibodies at diagnosis.

Of the total of 148 children, 19.6% had anti-HBe (Hepatitis B-e) antibodies at the time of diagnosis. Spontaneous seroconversion was predominantly recorded in those with chronic HBeAg- infection (73.9%), and 81.8% of those with chronic HBeAg- hepatitis were positive for anti-HBe antibodies. Among the 89 patients who did not undergo treatment, 44 (49.4%) developed spontaneous seroconversion in the HBe system. At the time of diagnosis, there were already 21 patients (23.6%) with anti-HBe antibodies present (from the total of treatment-naive patients) and 8 patients (13.6%) with anti-HBe antibodies (from those who later received treatment), making a total of 56 children with spontaneous HBe seroconversion (37.8%) out of 148—Figure 2.

The distribution of treatment-naive patients based on the age at which they experienced spontaneous seroconversion is exposed in Table 5. Most of the treatment-naïve patients who developed spontaneous seroconversion were 2 years old (9%), followed by ages 15 years (7.9%), 7 (4.5%), 8 (4.5%), 13 (4.5%).

The distribution of patients with spontaneous seroconversion based on the presence of giardiasis before conversion is exposed in Table 6. Among the 15 children and adolescents with spontaneously appearing anti-HBe antibodies out of the 44, an episode of giardiasis was detected 6 months to 1 year before seroconversion in 6 of them. A significant connection was persistent marked eosinophilia (on average, two times the normal value). In 7 other cases, giardiasis was not confirmed by parasitological examination, and for the remaining 21 children, specific documentation was not available, as they already had anti-HBe antibodies at the time of diagnosis.

### 3.2. Evolution under Treatment

The distribution of patients by antiviral treatment at each stage of the disease is exposed in Table 7. Regarding the administration of antiviral therapy based on the disease stage at diagnosis, most patients (64.4%) were in the stage of HBeAg+ hepatitis, 20.3% with HBeAg+ infection, 11.9% with HBeAg- infection, and only 3.4% in the stage of HBeAg- hepatitis. Most patients (84.7%) recommended for treatment were in the stage of intense viral replication.

The distribution of patients based on the administered treatment is exposed in Table 8. Most patients received Entecavir (14.2%), Interferon (13.5%), and Zeffix (Lamivudine) (10.8%). Ten patients (6.8%) were treated with Peginterferon, five patients (3.4%) received Tenofovir, and only one patient was administered Telbivudine (0.7%).

The prevalence of adverse reactions at treatment is exposed in Table 9. The distribution of each side effect in the study group is exposed in Table 10: 61% of patients receiving antiviral treatment experienced side effects, with fever being the most common (28.8%). This was followed by changes in the white blood cell count, such as leukopenia (10.2%) and neutropenia (3.4%), nausea/vomiting (8.5%), loss of appetite (6.8%), flu-like symptoms (headache, chills, abdominal pain, arthralgia).

The distribution of patients based on HBe seroconversion post-treatment is exposed in Table 11. and for each specific treatment in Table 12. As can be seen, out of the total number of patients who received antiviral treatment, 26 patients (44.1%) developed anti-HBe antibodies. Among those treated with Interferon, 50% experienced HBe seroconversion, while the other 50% did not develop HBe antibodies. For patients treated with Peginterferon, six of them (60%) developed HBe antibodies. In the case of patients treated with Entecavir, 15 (71.4%) did not undergo HBe seroconversion, while 6 (28.8%) developed antibodies. Telbivudine was administered to a single patient who experienced HBe seroconversion at the end of the treatment. For patients treated with Tenofovir, four out of five (80%) did not develop antibodies, while one had HBe seroconversion. Among patients treated with Lamivudine, out of a total of 16, only 11 (68.8%) exhibited HBe seroconversion.

## 4. Discussion

This longitudinal, retrospective study engages a substantial cohort of children of different ages assessed at a single medical center. It is designed to enhance our understanding of the natural progression and course of HBV infection within the pediatric population. Our study has revealed details intended to update the specialized literature in this field. Additionally, we have characterized the patients according to the new EASL nomenclature for hepatitis B.

### 4.1. Vertical Transmission

Hepatitis B virus (HBV) infection stands as a leading contributor to global morbidity and mortality. Typically, children contract the infection perinatally or in early childhood, leading to chronic hepatitis characterized by heightened viral replication and a phase of infection marked by low inflammation, with aminotransferase levels remaining normal or only slightly elevated [9]. Recent estimates suggest that the global prevalence of HBV infection among children under 5 years old stands at 1.3%, a notable decrease from the 4.7% reported during the 1980–2000 period [3]. However, despite advancements in immunization accessibility, approximately 2 million new HBV infections are reported annually within the 0–5 years age group [1]. Vertical transmission from mother to child during pregnancy or childbirth accounts for the majority of HBV infections, posing a heightened risk (over 90%) of chronic HBV infection lasting over six months [3].

In our study, the prevalence of HBV (Ag HBs+) was 22.6%. The prevalence in this cohort was higher than in the general population due to the patient population’s referral in the hospital setting within the Pediatric Gastroenterology specialty. From current data, there is no information regarding the prevalence of chronic viral hepatitis B infection in children at the national or regional level. Our study is the first important research both temporally (from 2011 to 2019) and in terms of being conducted at a regional university center, “Sf. Maria” Hospital, which is a major pediatric hospital in Romania, with patients referred from all counties of the Northeast—region of Romania and beyond.

### 4.2. Clinical Manifestations

Most patients in our study were diagnosed at the stage of chronic viral hepatitis B HBeAg positive, followed by HBV infection HBeAg positive. In the initial stage of HBV infection, typically acquired at birth, the virus exhibits high replication levels, characterized by the presence of HBeAg and extremely elevated HBV DNA concentrations in the blood, often surpassing 100 million or even 1 billion IU/mL. This stage, known as the immune-tolerance phase (old nomenclature), commonly persists for 10 to 30 years, during which the natural elimination of HBeAg from the body is unlikely. Approximately 3–5% of children with chronic HBV infection develop cirrhosis, and a small percentage, ranging from 0.01 to 0.03%, develop hepatocellular carcinoma [4].

Although many children may be completely asymptomatic, the clinical picture depends on the age at diagnosis, sex, mode of viral transmission, viral genotype, or the patient’s immune status. Almost two-thirds of the patients in our study group were symptomatic, but the majority exhibited nonspecific symptoms such as loss of appetite (16.2%), fatigue, and digestive disturbances. Some of them presented also for respiratory tract infections (6.8%) or for abdominal pain (12.8%). While almost half of the patients with HBeAg+ infection exhibited signs or symptoms, three-quarters of those with chronic HBeAg+ hepatitis displayed clinical symptoms and signs. Children with HBeAg- or HBeAg+ infection complained mostly of loss of appetite, while abdominal pain and hepatomegaly were more common in HBeAg- hepatitis. Other clinical manifestations can be found in approximately 25% of cases through skin signs such as palmar–plantar erythema, erythema nodosum, vasculitis, and joint signs such as arthralgia, arthritis, or diaphyseal pain. There may also be muscle signs like myositis, myalgia, muscle hypotonia, renal signs such as membranoproliferative or membranous glomerulonephritis, and hematological signs—hemolytic or macrocytic anemia, thrombocytopenia, leukopenia, hemorrhagic syndromes [10].

### 4.3. Spontaneous Seroconversion in HBe System

Spontaneous seroconversion in our study (37.8%) was predominantly recorded in those with chronic HBeAg- infection (73.9%), while most patients with chronic HBeAg- hepatitis were positive for anti-HBe antibodies. Unlike children with perinatal infection, children who acquire the infection horizontally often experience spontaneous HBeAg seroconversion. It is known that HBe seroconversion is more common in the pediatric population than in adults. In two Italian studies focused on children primarily infected via horizontal transmission, the annual rate of spontaneous HBeAg seroconversion ranged from 14 to 16% during the initial decade of follow-up [11]. In patients with HBeAg+ infection, seroconversion occurs in the HBe system, and ALT may increase before clearance and remain altered up to 6–12 months after seroconversion. It is estimated that in the first 3 years of this period, the occurrence rate of anti-HBe antibodies is 2% and reaches 15% in the first 20 years [7].

An American study found that 25% of Asian Americans experienced spontaneous HBeAg seroconversion by the age of 17, and 50% of Asian Americans had such an experience by the age of 24. Similarly, in a Canadian study, 37% of the enrolled children, of whom 80% were Asian and 59% had perinatal transmission, underwent spontaneous HBeAg seroconversion at the average age of 14.5 years [12,13].

Nevertheless, HBeAg seroconversion, typically indicative of a favorable prognosis, is not always a permanent change. Even in individuals who test positive for anti-HBe antibodies, the low-level replication of HBV can persist, and transitions in HBe status occur in up to 10% of viral carriers, potentially heightening the risk of complications. Viral replication, evidenced by HBV DNA positivity, is also observed in cases of HBeAg-negative mutant infection and remains a crucial parameter to consider. Presently, available data do not indicate any distinct prognosis for patients affected by this type of infection [14]. The inactive hepatitis phase, or HBeAg-negative chronic HBV infection, is identified by anti-HBe antibodies, undetectable or low HBV DNA levels, and normal ALT levels. Nonetheless, patients may still experience histologic inflammation and/or fibrosis, underscoring the need for continued monitoring and potential intervention [7].

### 4.4. Antiviral Treatment

We noted that most patients recommended for treatment were in the stage of intense viral replication (HBeAg+). Most administered antivirals for patients with VHB infection and VHB hepatitis were Entecavir, followed by Interferon and Zeffix. Other antivirals were Peginterferon, Tenofovir, and Telbivudine. Less than half of patients receiving antivirals developed anti-HBe antibodies. Favorable results for Lamivudine (Zeffix) were observed in our study, with 68.8% of children who underwent treatment with this antiviral developing anti-HBe antibodies. Entecavir was administered to 21 children, with 6 of them developing HBe seroconversion. Among those treated with PegInterferon (10), 6 developed anti-HBe antibodies, while for those treated with IFN alpha, the proportions were 50–50%. These results were recorded up until the time of the last hospital presentation due to a lack of compliance for further recordings. Thus, Peginterferon led to the highest rate of HBe seroconversion (60%), followed by Interferon (50%), while only 28.8% of patients receiving Entecavir developed HBe antibodies. The primary objectives of antiviral treatment for children with chronic HBV infection include suppressing viral replication and preventing disease progression to cirrhosis and hepatocellular carcinoma, although these complications are uncommon in children [9]. The management of chronic viral B hepatic infection is guided by certain parameters, such as the ALT level, HBV DNA level, presence or absence of HBeAg, histological appearance of the liver, patient’s personal history as well as family history concerning HCC (hepatocellular carcinoma), and presence of other liver diseases. It is recommended that the ALT level for initiating antiviral treatment should be at least 1.5 times the normal value/>60 IU/L [4]. Peginterferon α (PEG-IFN) was reported to be an effective antiviral in the treatment of children and adults with chronic hepatitis B (CHB) infection [15]. Kim et al. (2009) reported the development of anti-HBe antibodies after antiviral treatment (Lamivudine) in only 26.5% of HVB-positive children. It was found that the age at which antiviral treatment started was the only factor associated with HBs Ag elimination (mean age of 5.1 ± 4.3 years for patients with seroconversion versus 7.9 ± 4.9 years for patients without seroconversion, *p* = 0.006) [16]. He et al. analyzed the response to antiviral treatment with Interferon (26) and Entecavir (44) of 70 treatment-naive children over 48 weeks [17]. The results consisted of higher rates of serological response to HBe antigen and HBs antigen but lower rates of virological and biochemical response to Pegylated Interferon compared to Entecavir at week 48. Additionally, the high pre-treatment level of IL-18 (Interleukin-18) was significantly associated with serological response to HBe antigen and remained the sole independent predictor of anti-HBe antibodies after adjustment for other important factors. The data corroborate with those from our study, showing that more than 50% of those treated with Pegylated Interferon exhibited HBe seroconversion, whereas in those treated with Entecavir, the viral load significantly decreased in 10 out of 13 cases recorded. Komatsu et al. found that the use of Pegylated Interferon in children chronically infected with HBV genotype C at doses of 180 μg/1.73 m^2^ or higher (median, 287 μg/1.73 m^2^) resulted in a favorable serological response [18]. Out of 12 treated children, 67% developed anti-HBe antibodies, and 8% developed anti-HBs antibodies. Also, even though entecavir was approved in 2014 for use in children aged at least 2 years in the form of an oral solution, it is not available in this form in our country, which is why it was not possible to administer it to young children.

It has been demonstrated that IFNα-2b accelerates the onset of seroconversion in the HBe system, even if the patient does not respond to treatment during therapy, but will develop anti-HBe antibodies more rapidly than if they had not received antivirals [19]. HBe seroconversion can occur anytime up to 1 year after completing IFN-alpha therapy; therefore, a patient is not declared a non-responder to treatment, nor is another antiviral introduced within 6–12 months after the end of the first treatment, unless signs of decompensation appear. The HBe seroconversion rate reaches 32% among patients treated with pegylated IFN alfa-2a, compared to 18% with lamivudine, 12% with adefovir, 21% with entecavir, and 23% with telbivudine [19]. These are important data that can be correlated with the results from the analysis of our cohort, highlighting the increased rate of HBe seroconversion for pegylated IFN.

### 4.5. Treatment Side Effects

More than half of the patients receiving antiviral treatment (61%) reported side effects. The most frequent side effect was fever (28.8%), followed by changes in the white blood cell count (leukopenia and neutropenia), nausea/vomiting, loss of appetite. While a cautious approach is often advised in pediatric cases, various therapies are available, allowing for diverse therapeutic strategies [9]. With IFN treatment, possible adverse reactions such as fever, flu-like symptoms, neutropenia, gastrointestinal and behavioral disturbances, and weight loss are known. These are transient and resolve after treatment cessation [20]. In NA (nucleoside analog) treatment, exacerbations are encountered through increased ALT, gastrointestinal distress, asthenia, headache, peripheral neuropathy, lactic acidosis, pancreatitis, and myalgia [21]. Therefore, a conservative approach to treatment initiation in children is recommended due to the side effects of these antivirals. These side effects were also reported in our study, thus reinforcing the data from the literature and highlighting the need for their reporting for a safe progression of patients.

### 4.6. Correlations between HBe Seroconversion and Parasitosis

In our study, we assessed the possibility of correlations between HBe seroconversion and intestinal parasitosis—15 out of 16 patients with giardiasis developed spontaneous HBe seroconversion at 6–12 months after parasitosis. The literature did not mention the existence of such a correlation despite the presence of eosinophilia in both pathologies. Giardia lamblia is the most prevalent gastrointestinal parasite in developed countries. It leads to both epidemic and endemic gastrointestinal disturbances, including diarrhea. Patients with compromised immune systems, such as those diagnosed with common variable hypogammaglobulinemia or lymphoproliferative diseases affecting the gastrointestinal tract, appear to be unusually vulnerable to giardiasis [22]. Additionally, their infections often prove challenging to treat. Nurmatova et al., in a recent study, suggest that the infection with giardiasis and the extension of both the hepatic pathological process and the overall infectious process in children with chronic viral hepatitis B are closely linked [23]. These findings strongly highlight the necessity of promptly eradicating lamblia in cases of liver damage. Furthermore, when administering specific medications, their hepatotoxicity, bioavailability, and effectiveness should be carefully considered. Regardless, there could be a correlation between the two pathologies; this remains to be closely observed in detail in larger studies.

## 5. Conclusions

Pediatric patients with chronic viral B infection may experience a slow progression of the disease without specific signs and symptoms for an extended period, especially if diagnosed at an early age, such as infancy or early childhood. The primary limitations of this study include ending the follow-up at 18 years of age, which restricts the available patient data, such as seroconversion rates. Moreover, some patients did not have consistent monitoring and had long gaps between hospital visits, hindering a more thorough follow-up of the cases. However, it is important to seek early medical consultation for monitoring and treatment appropriate to the age and stage of the disease. We believe that this study is useful for the new data; it provides and contributes to the current literature in the field.

## Figures and Tables

**Table 1:** Natural history of HBV infection [8].

New EASL Nomenclature	Old Nomenclature	Viral Replication	Risk of HCC	ALT Values
HBeAg+ Chronic HBV Infection	Immunotolerant	High Replicative Phase	Low	Low
HBeAg+ Chronic Hepatitis B	Immuno-clearance/Active Immune Phase	Moderately Replicative Phase (HBeAg+/-)	Increased	High
HBeAg- Chronic HBV Infection	Inactive Carrier	Low Replicative Phase	Low	Low
HBeAg- Chronic Hepatitis HBV	Chronic Hepatitis HBeAg	Moderately Replicative Phase HBeAg-	Increased	High

**Table 2:** Distribution of patients in each phase of the infection by age.

Age Group (Years)	HBeAg- Hepatitis	HBeAg+ Hepatitis	HBeAg- Infection	HBeAg+ Infection	Total
Pearson Chi-Square = 12,189/*p* = 0.007 **	*n*%	*n*%	*n*%	*n*%	*n*%
0–1 year	2 (18.2%)	19 (26.0%)	-	9 (22.0%)	30 (20.3%)
2–5 years	2 (18.2%)	17 (23.3%)	8 (34.8%)	12 (29.3%)	39 (26.4%)
6–10 years	3 (27.3%)	14 (19.2%)	6 (26.1%)	11 (26.8%)	34 (23.0%)
11–14 years	1 (9.1%)	16 (21.9%)	6 (26.1%)	5 (12.2%)	28 (18.9%)
15–18 years	3 (27.3%)	7 (9.6%)	3 (13.0%)	4 (9.8%)	17 (11.5%)
Total	11 (100.0%)	73 (100.0%)	23 (100.0%)	41 (100.0%)	148 (100.0%)
Patients related to group (%)	7.4%	49.3%	15.5%	27.7%	100%

** refers that the test is statistically significant and indicates a likely association between the age and the phases of infection.

**Table 3:** Prevalence of clinical manifestation in study group.

Disease Stage at DG	HBeAg+ Infection	HBeAg- Infection	HBeAg+ Hepatitis	HBeAg- Hepatitis	Total
Pearson Chi-Square = 12,189/*p* = 0.007 **	*n*%	*n*%	*n*%	*n*%	*n*%
Clinical Manifestation NO	23 (56.1%)	11 (47.8%)	18 (24.7%)	4 (36.4%)	56 (37.8%)
Clinical Manifestation YES	18 (43.9%)	12 (52.2%)	55 (75.3%)	7 (63.6%)	92 (62.2%)
Total	41 (100.0%)	23 (100.0%)	73 (100.0%)	11 (100.0%)	148 (100.0%)

** refers that the test is statistically significant and indicates a likely association between the clinical manifestations and the phases of infection.

**Table 4:** Clinical manifestations in study group.

Clinical Manifestations	HBeAg+ Infection *n*, %	HBeAg- Infection *n*, %	HBeAg+ Hepatitis *n*, %	HBeAg- Hepatitis *n*, %	Total *n*, %	Pearson Chi-Square/*p*-Value
Vomiting	-	2 (8.7%)	4 (5.5%)	-	6 (4.1%)	3.852/0.278
Loss of appetite	2 (4.9%)	4 (17.4%)	17 (23.3%)	1 (9.1%)	24 (16.2%)	7.001/0.072
Physical weakness	3 (7.3%)	1 (4.3%)	1 (1.4%)	1 (9.1%)	6 (4.1%)	3.197/0.362
Semi consistent stools	1 (2.4%)	1 (4.3%)	3 (4.1%)	1 (9.1%)	6 (4.1%)	0.998/0.802
Abdominal pain	3 (7.3%)	3 (13.0%)	11 (15.1%)	2 (18.2%)	19 (12.8%)	1.723/0.632
Hepatomegaly	1 (2.4%)	-	15 (20.5%)	2 (18.2%)	18 (12.2%)	11.991/0.007 **
Odynophagia	1 (2.4%)	-	6 (8.2%)	1 (9.1%)	8 (5.4%)	3.442/0.328
Cough	1 (2.4%)	2 (8.7%)	6 (8.2%)	1 (9.1%)	10 (6.8%)	1.693/0.638
Rhinorrhea/pharyngeal congestion	4 (9.8%)	1 (4.3%)	4 (5.5%)	1 (9.1%)	10 (6.8%)	1.081/0.782
Epistaxis	3 (7.3%)	-	3 (4.1%)	-	6 (4.1%)	2.559/0.465
Fever	2 (4.9%)	1 (4.3%)	4 (5.5%)	-	7 (4.7%)	0.647/0.886
Fatigue	1 (2.4%)	2 (8.7%)	2 (2.7%)	-	5 (3.4%)	2.579/0.461
Others	5 (12.2%)	4 (17.4%)	14 (19.2%)	3 (27.3%)	26 (17.6%)	1.664/0.645

** refers that the test is statistically significant and indicates a likely association between the hepatomegaly and the phases of infection.

**Table 5:** Spontaneous HBe seroconversion related to age.

Age	*n*	%
5 months	1	1.1
1, 2 years	2	2.2
2 years	8	9.0
3 years	2	2.2
5 years	1	1.1
6 years	2	2.2
7 years	4	4.5
8 years	4	4.5
9 years	2	2.2
10 years	1	1.1
11 years	1	1.1
12 years	2	2.2
13 years	4	4.5
14 years	2	2.2
15 years	7	7.9
16 years	1	1.1

**Table 6:** Distribution of patients with spontaneous seroconversion based on the presence of giardiasis (lamblia) pre-conversion.

HBe Spontaneous Seroconversion	Lamblia Pre- Seroconversion No (*n*, %)	Lamblia Pre- Seroconversion Yes (*n*, %)	Lamblia Pre- Seroconversion Unknown (*n*, %)	Total (*n*, %)
Pearson Chi-Square = 1.791/*p* = 0.408				
NO	0 (0.0%)	1 (6.3%)	-	1 (2.3%)
YES	7 (100%)	15 (93.8%)	21 (100%)	43 (97.7%)
Total	7 (100%)	16 (100%)	21 (100%)	44 (100%)

**Table 7:** Distribution of patients by antiviral treatment at each stage of the disease.

Disease Stage at DG	Antiviral Treatment No (*n*, %)	Antiviral Treatment Yes (*n*, %)	Total (*n*, %)
Pearson Chi-Square = 9.456/*p* = 0.024			
HBeAg+ Infection	29 (32.6%)	12 (20.3%)	41 (27.7%)
HBeAg- Infection	16 (18.0%)	7 (11.9%)	23 (15.5%)
HBeAg+ Hepatitis	35 (39.3%)	38 (64.4%)	73 (49.3%)
HBeAg- Hepatitis	9 (10.1%)	2 (3.4%)	11 (7.4%)
Total	89 (100%)	59 (100%)	148 (100%)

**Table 8:** Distribution of patients based on the administered treatment.

Antiviral Treatment	*n*	%
Interferon	20	13.5
Peginterferon	10	6.8
Entecavir	21	14.2
Telbivudine	1	0.7
Tenofovir	5	3.4
Zeffix	16	10.8

**Table 9:** Distribution of patients based on the presence of side effects at treatment.

Side Effects	*n*	%
No	23	39%
Yes	36	61%
Total	59	100%

**Table 10:** Distribution of patients based on clinical manifestations in adverse reactions.

Clinical Manifestations	*n*	%
Fever	17	28.8
Leukopenia	6	10.2
Nausea/vomiting	5	8.5
Loss of appetite	4	6.8
Osteopenia	4	6.8
Abdominal pain	4	6.8
Chills	3	5.1
Headache	3	5.1
Neutropenia	2	3.4
Hypertriglyceridemia	2	3.4
Psychomotor agitation	2	3.4
Arthralgia	2	3.4
Cardiovascular disorders	2	3.4
Epistaxis/gingival bleeding	1	1.7
Fracture	1	1.7
Hypothyroidism	1	1.7
Lymphopenia	1	1.7
Myalgia	2	3.4
Thrombocytopenia	1	1.7
Urticaria	1	1.7
Vertigo	1	1.7

**Table 11:** Distribution of patients based on HBe seroconversion after treatment.

HBe Seroconversion Post-Treatment	*n*	%
No	33	55.9
Yes	26	44.1
Total	59	100

**Table 12:** Distribution of HBe seroconversion in relation to antiviral medication.

Treatment	Intron A	PegIFN	Entecavir	Telbivudine	Tenofovir	Zeffix
HBe seroconversion post-treatment	50%	60%	28.8%	100%	20%	68.8%
Nr patients	10	6	6	1	1	11

## Data Availability

The original contributions presented in the study are included in the article, further inquiries can be directed to the corresponding author.
